# Automated detection of nonmelanoma skin cancer using digital images: a systematic review

**DOI:** 10.1186/s12880-019-0307-7

**Published:** 2019-02-28

**Authors:** Arthur Marka, Joi B. Carter, Ermal Toto, Saeed Hassanpour

**Affiliations:** 1 Dartmouth Geisel School of Medicine, Box 163, Kellogg Building, 45 Dewey Field Road, Hanover, NH USA; 2 Section of Dermatology, Dartmouth-Hitchcock Medical Center, Lebanon, NH USA; 3 Department of Surgery, Dartmouth Geisel School of Medicine, Hanover, NH USA; 4 Department of Computer Science, Worcester Polytechnic Institute, Worcester, MA USA; 5 Department of Biomedical Data Science, Dartmouth College, Lebanon, NH USA

**Keywords:** Nonmelanoma skin cancer, Squamous cell carcinoma, Basal cell carcinoma, Artificial intelligence, Machine learning, Computer-aided diagnosis, Image analysis

## Abstract

**Background:**

Computer-aided diagnosis of skin lesions is a growing area of research, but its application to nonmelanoma skin cancer (NMSC) is relatively under-studied. The purpose of this review is to synthesize the research that has been conducted on automated detection of NMSC using digital images and to assess the quality of evidence for the diagnostic accuracy of these technologies.

**Methods:**

Eight databases (PubMed, Google Scholar, Embase, IEEE Xplore, Web of Science, SpringerLink, ScienceDirect, and the ACM Digital Library) were searched to identify diagnostic studies of NMSC using image-based machine learning models. Two reviewers independently screened eligible articles. The level of evidence of each study was evaluated using a five tier rating system, and the applicability and risk of bias of each study was assessed using the Quality Assessment of Diagnostic Accuracy Studies tool.

**Results:**

Thirty-nine studies were reviewed. Twenty-four models were designed to detect basal cell carcinoma, two were designed to detect squamous cell carcinoma, and thirteen were designed to detect both. All studies were conducted in silico. The overall diagnostic accuracy of the classifiers, defined as concordance with histopathologic diagnosis, was high, with reported accuracies ranging from 72 to 100% and areas under the receiver operating characteristic curve ranging from 0.832 to 1. Most studies had substantial methodological limitations, but several were robustly designed and presented a high level of evidence.

**Conclusion:**

Most studies of image-based NMSC classifiers report performance greater than or equal to the reported diagnostic accuracy of the average dermatologist, but relatively few studies have presented a high level of evidence. Clinical studies are needed to assess whether these technologies can feasibly be implemented as a real-time aid for clinical diagnosis of NMSC.

**Electronic supplementary material:**

The online version of this article (10.1186/s12880-019-0307-7) contains supplementary material, which is available to authorized users.

## Background

Nonmelanoma skin cancer (NMSC) is by far the most common malignancy in humans, with an estimated 3,300,000 annual cases in the United States alone [1]. Over 95% of NMSC cases are basal cell carcinoma (BCC) and cutaneous squamous cell carcinoma (CSCC) [2], both of which may be readily identified through visual inspection by a skilled dermatologist. However, multiple benign lesions can mimic these cancers, resulting in unnecessary morbidity through invasive biopsies and treatments. For example, the SCREEN study, which included 15,983 biopsies performed in 360,288 adults for suspected skin cancer, found that approximately five biopsies had to be performed to detect one malignant skin lesion of any type [3].

The use of artificial intelligence (AI) as a diagnostic aid is a growing trend in dermatology. These systems generally utilize some form of machine learning (ML), which is a subset of AI involving methods that enable machines to make predictions based on their prior data and experiences. In contrast to conventional models that are explicitly programmed to handle a static set of cases, ML models can derive their own generalizations based on a training set and perform accurately in novel scenarios.

Automated classification of NMSC has been achieved through a variety of modalities, such as Raman spectroscopy, optical coherence tomography, and electrical impedance [4–6]. However, the simplest modality is digital photography, often enhanced by a dermatoscope. Given the near ubiquitous use of digital cameras and dermatoscopes in dermatologic practice, digital image-based ML models have the greatest potential for clinical implementation and are thus the focus of this review.

Previous reviews of artificial intelligence and skin cancer have focused on melanoma [7–9]. To our knowledge, the present study represents the first systematic review of automated detection of NMSC using digital image analysis. The objectives of this study are to identify which digital image-based ML models have been used to diagnose BCC and CSCC and to assess the evidence for their diagnostic accuracy.

## Methods

The review was registered in the PROSPERO international prospective register of systematic reviews (Record number: CRD42017060981) and follows the guidelines of the PRISMA Statement. The PRISMA checklist is included in Additional file 1.

### Search strategy

Articles were identified from searches of PubMed, Google Scholar, Embase, IEEE Xplore, SpringerLink, ScienceDirect, Web of Science, and the ACM Digital Library using Boolean operators with no search restrictions. Syntactic modifications were made to accommodate the parameters of the databases while preserving the logic of the search string. The following search string was used:

(Association rule OR Automat* detection OR Classification OR Classifier OR Computer-aided OR Computer-assisted OR Computer vision OR Cluster OR Bayes* OR Deep learning OR Decision tree OR Ensemble OR (Feature AND (extraction OR selection)) OR Genetic algorithm OR Inductive logic OR KNN OR K-means OR Machine learning OR Neural network OR Pattern recognition OR Regression OR Random forest OR Support vector) AND (Basal cell carcinoma OR Squamous cell carcinoma) AND (Skin OR Cutaneous OR Dermatolog*) AND (Dermatoscop* OR Dermoscop* OR Image OR Photograph* OR Picture)

Machine learning terms were taken from textbooks on machine learning and represent the most commonly used models [10, 11]. Note that the search string contained terms to exclude studies of noncutaneous cancers.

### Study selection

**Fig. 1:**
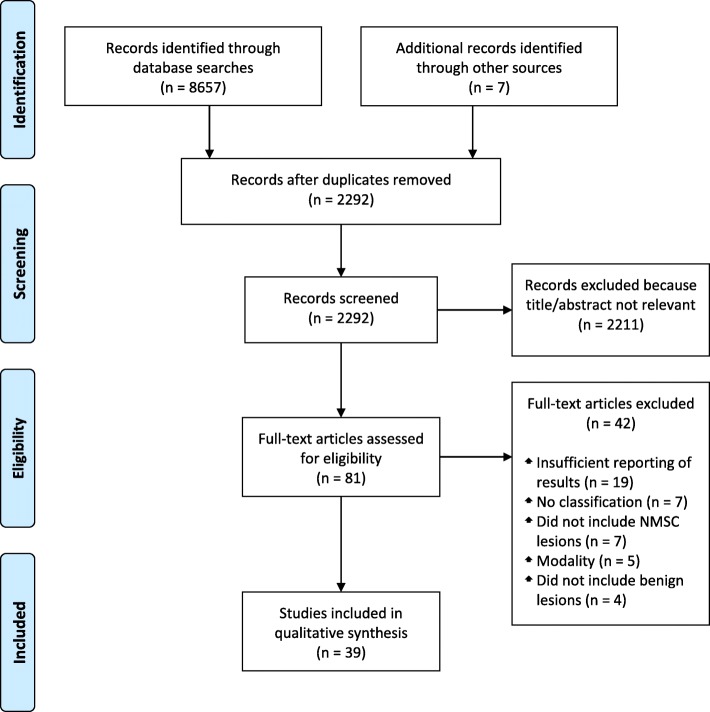
PRISMA flow diagram of study selection. Abbreviation: PRISMA, Preferred Reporting Items for Systematic Reviews and Meta-Analyses

Two investigators extracted data, and results were cross-validated at each step of the selection protocol. Studies were included according to the following selection criteria: (i) classification of NMSC versus benign lesion, (ii) machine learning method, (iii) digital image modality, and (iv) publication in English. Several studies met these criteria but were excluded because they involved classification of both melanoma and NMSC but did not report NMSC-specific performance metrics. We have reported only the NMSC-specific results in studies that classified both melanoma and NMSC. Furthermore, while some studies tested multiple models, we have reported only the model that achieved the highest NMSC-specific performance in each study. References cited in the studies identified from the literature databases served as an additional source of included articles. The selection protocol has been illustrated in the PRISMA flow diagram in Fig. 1.

### Quality assessment

The overall quality of each included study was rated according to a modified version of the Levels of Evidence from *The Rational Clinical Examination*, shown in Table 1 [12]. The original rating scheme specifies the highest level of evidence as blinded, independent studies that compare the diagnostic tool in question against a criterion standard in a large, consecutive sample of patients suspected of having the target condition. Given that all of the included studies were conducted in silico, the interpretation of this definition was modified as follows: (i) blinding was equated to no overlap of images between training and test sets, (ii) independence was equated to avoiding the selective use of images containing features of interest in the test set, and (iii) test sets were considered consecutive if they were obtained from a clinic or set of clinics in which all lesions for which there was any suspicion of malignancy were included. The reporting quality, risk of bias, and applicability of each study was further assessed using the Quality Assessment of Diagnostic Accuracy Studies (2nd edition, QUADAS-2) tool [13].

**Table 1:** Levels of evidence^a^

Level of evidence	Definition
1	Independent, blinded comparison of the classifier with a biopsy-proven standard among a large number of consecutive lesions suspected of being the target condition
2	Independent, blinded comparison of the classifier with a biopsy-proven standard among a small number of consecutive lesions suspected of being the target condition
3	Independent, blinded comparison of the classifier with a biopsy-proven standard among non-consecutive lesions suspected of being the target condition
4	Non-independent comparison of the classifier with a biopsy-proven standard among obvious examples of the target condition plus benign lesions
5	Non-independent comparison of the classifier with a standard of uncertain validity

^a^Modified from Simel and Rennie [12]

## Results

The database searches returned 8657 total results, of which 2285 were found to be unique after de-duplication. The titles and abstracts of the unique studies were reviewed, and 2211 articles were deemed irrelevant and excluded. Manual review of the references cited within the remaining 74 studies identified seven additional studies of potential relevance, for a total of 81 studies, which were read in their entirety for assessment of eligibility. Of these 81 studies, 42 were excluded due to disqualifying methodologies or insufficient reporting of results. Thus, a total of 39 studies were ultimately included in the review. The characteristics of the included studies are shown in Table 2.

**Table 2:** Overview of literature search

Source	Target NMSC	Digital Image Modality	Database	Algorithm	Outcome	Quality Rating^a^
Abbas, 2016 [14]	BCC; CSCC	Dermoscopic	30 BCCs, 30 CSCCs, 300 various other lesions (30% of dataset used for training and 70% for testing)^b^	ANN	AUROC: 0.92, (BCC), 0.95 (CSCC); Sensitivity: 97% (BCC), 97% (CSCC); Specificity: 68% (BCC), 80% (CSCC)	5
Ballerini, 2012 [43]	BCC; CSCC	Non-dermoscopic	239 BCCs, 88 CSCCs, 633 benign lesions (3-fold cross-validation)	k-NN^c^	Accuracy: 89.7%^d^; Sensitivity: 89.9%^d^; Specificity: 89.6%^d^	3
Chang 2013 [15]	BCC; CSCC	Non-dermoscopic	110 BCCs, 20 CSCCs, 639 various other lesions (leave-one-out cross-validation)^b^	MSVM	Sensitivity: 90% (BCC), 80% (CSCC)	2
Cheng, 2011 [34]	BCC	Dermoscopic	59 BCCs, 152 benign lesions (leave-one-out cross-validation)	ANN	AUROC: 0.967	4
Cheng, 2012 [36]	BCC	Dermoscopic	263 BCCs, 226 benign lesions (10-fold cross-validation)	ANN^c^	AUROC: 0.846	4
Cheng, 2013 [37]	BCC	Dermoscopic	35 BCCs, 79 benign lesions (leave-one-out cross-validation)	ANN	AUROC: 0.902	4
Cheng, 2013 [40]	BCC	Dermoscopic	350 BCCs, 350 benign lesions (10-fold cross-validation)	ANN^c^	AUROC: 0.981	2
Choudhury, 2015 [24]	BCC; CSCC	Dermoscopic; Non-dermoscopic	359 BCCs, CSCCs, MMs, and AKs (40 from each class randomly chosen for training; remainder used for testing)^b^	MSVM^c^	Accuracy: 94.6% (BCC), 92.9% (CSCC)	5
Chuang, 2011 [33]	BCC	Non-dermoscopic	84 BCCs, 235 benign lesions (3-fold cross-validation)	ANN	Accuracy: 95.0%; Sensitivity: 94.4%; Specificity: 95.2%	3
Dorj, 2018 [25]	BCC; CSCC	Non-dermoscopic	Training: 728 BCCs, 777 CSCCs, 768 MMs, and 712 AKs; Testing: 193 BCCs, 200 CSCCs, 190 MMs, and 185 AKs	ANN	Accuracy: 91.8% (BCC), 95.1% (CSCC); Sensitivity: 97.7% (BCC), 96.9% (CSCC); Specificity: 86.7% (BCC), 94.1% (CSCC)	5
Esteva, 2017 [16]	BCC; CSCC	Dermoscopic; Non-dermoscopic	Training: 127463 various lesions (9-fold cross-validation); Testing: 450 BCCs and CSCCs, 257 SKs	ANN	AUROC: 0.96	3
Ferris, 2015 [17]	BCC; CSCC	Dermoscopic	11 BCCs, 3 CSCCs, 39 MMs, 120 benign lesions (half used for training and half for testing)	Decision forest classifier	Sensitivity: 78.6%	2
Fujisawa, 2018 [18]	BCC; CSCC	Non-dermoscopic	Training: 974 BCCs, 840 CSCCs, 3053 various other lesions; Testing: 249 BCCs, 189 CSCCs, 704 various other lesions^b^	ANN	Sensitivity: 80.3% (BCC), 82.5% (CSCC)	3
Guvenc, 2013 [47]	BCC	Dermoscopic	68 BCCs, 131 benign lesions (no cross-validation)	Logistic regression	Accuracy: 96.5%; AUROC: 0.988	4
Han, 2018 [23]	BCC; CSCC	Non-dermoscopic	Training: 19398 various lesions; Testing: 499 BCCs, 211 CSCCs, 2018 various other lesions^b,e^	ANN	AUROC: 0.96 (BCC), 0.91 (CSCC); Sensitivity: 88.8% (BCC), 90.2% (CSCC); Specificity: 91.7% (BCC); 80.0% (CSCC)	3
Immagulate, 2015 [31]	BCC; CSCC	Non-dermoscopic	100 BCCs, 100 CSCCs, 100 AKs, 100 SKs, 100 nevi (10-fold cross-validation)	MSVM^c^	Accuracy: 93%	5
Kefel, 2012 [49]	BCC	Dermoscopic	49 BCCs, 153 benign lesions (leave-one-out cross-validation)	ANN	AUROC: 0.925	4
Kefel, 2016 [38]	BCC	Dermoscopic	Training: 100 BCCs, 254 benign lesions; Testing: 304 BCCs, 720 benign lesions	Logistic regression	AUROC: 0.878	2
Kharazmi, 2011 [48]	BCC	Dermoscopic	299 BCCs, 360 benign lesions (no cross-validation)	Random forest classifier	AUROC: 0.903	4
Kharazmi, 2016 [50]	BCC	Dermoscopic	299 BCCs, 360 benign lesions (no cross-validation)	Random forest classifier	AUROC: 0.965	4
Kharazmi, 2017 [51]	BCC	Dermoscopic	Training: 149 BCCs, 300 benign lesions; Testing: 150 BCCs, 300 benign lesions	ANN	AUROC: 0.911; Sensitivity: 85.3%; Specificity: 94.0%	3
Kharazmi, 2018 [52]	BCC	Dermoscopic	295 BCCs; 369 benign lesions (10-fold cross-validation)	Random forest classifier	AUROC: 0.832; Sensitivity: 74.9%; Specificity: 77.8%	3
Lee 2018 [29]	BCC	Non-dermoscopic	Training: 463 BCCs, 1914 various lesions; Testing: 51 BCCs, 950 various lesions^b^	ANN	Sensitivity: 91%	3
Maurya, 2014 [19]	BCC; CSCC	Dermoscopic; Non-dermoscopic	84 BCCs, 101 CSCCs, 77 MMs, 101 AKs (75 from each class used for training; remainder used for testing)^b^	MSVM	Accuracy: 83.3% (BCC), 84.1% (CSCC)	5
Mishra, 2017 [39]	BCC	Dermoscopic	305 BCCs, 718 benign lesions (leave-one-out cross-validation)	Logistic regression	Accuracy: 72%^f^	3
Møllersen, 2015 [30]	BCC; CSCC	Dermoscopic	Training: 37 MMs, 169 various lesions^g^; Testing: 71 BCCs, 7 CSCCs, 799 various lesions^b^	Hybrid model of linear and quadratic classifiers^c^	Sensitivity: 100%; Specificity: 12%	2
Shakya, 2012 [41]	CSCC	Dermoscopic	53 CSCCs, 53 SKs (no cross-validation)	Logistic regression	AUROC: 0.991	4
Shimizu, 2014 [20]	BCC	Dermoscopic	69 BCCs, 105 MMs, 790 benign lesions (10-fold cross-validation)^b^	Layered model of linear classifiers^c^	Sensitivity: 82.6%	3
Shoieb, 2016 [26]	BCC	Non-dermoscopic	Training: 84 NMSC, 119 MMs; Testing: 64 BCC, 72 MM, 74 eczema, 31 impetigo	MSVM	Accuracy: 96.2%; Specificity: 96.0%; Sensitivity: 88.9%	5
Stoecker, 2009 [35]	BCC	Dermoscopic	42 BCCs, 168 various lesions(leave-one-out cross-validation)^b^	ANN	AUROC: 0.951	2
Sumithra, 2015 [21]	CSCC	Non-dermoscopic	31 CSCCs, 31 MMs, 33 SKs, 26 bullae, 20 shingles (70% used for training; remainder used for testing)^b^	Hybrid model of MSVM and k-NN classifiers^c^	F-measure: 0.581	5
Upadhyay, 2018 [27]	BCC; CSCC	Non-Dermoscopic	239 BCCs, 88 CSCCs, 973 various lesions (24 from each class used for training; remainder used for testing)^b^	ANN	Accuracy: 96.6% (BCC), 81.2% (CSCC); Sensitivity: 96.8% (BCC), 80.5% (CSCC)	3
Wahab, 2003 [32]	BCC	Non-Dermoscopic	54 BCCs, 54 DLE, 54 AV (34 from each class used for training; remainder used for testing)	ANN	Sensitivity: 90%	5
Wahba, 2017 [22]	BCC	Dermoscopic	29 BCCs, 27 nevi (46 total used for training and 10 for testing)	MSVM	Accuracy: 100%; Sensitivity: 100%; Specificity: 100%	5
Wahba, 2018 [42]	BCC	Dermoscopic	300 BCCs, 300 MMs, 300 nevi, 300 SKs (fivefold cross-validation)^b^	MSVM	AUROC: Sensitivity: 100%; Specificity: 100%	3
Yap, 2018 [28]	BCC	Dermoscopic; Non-Dermoscopic	647 BCCs, 2270 various lesions (fivefold cross-validation)^b^	ANN	Accuracy: 91.8%; Sensitivity: 90.6%; Specificity: 92.3%	3
Zhang, 2017 [44]	BCC	Dermoscopic	132 BCCs, 132 nevi, 132 SKs, 132 psoriasis (80% used for training; remainder used for testing)	ANN	Accuracy: 92.4%^d^; Sensitivity: 85%^d^; Specificity: 94.8%^d^	3
Zhang, 2018 [45]	BCC	Dermoscopic	132 BCCs, 132 nevi, 132 SKs, 132 psoriasis (10-fold cross-validation)	ANN	Accuracy: 94.3%^d^; Sensitivity: 88.2%^d^; Specificity: 96.1%^d^	3
Zhou, 2017 [46]	BCC	Dermoscopic	Training: 154 BCCs, 10,262 benign lesions; Testing: 50 BCCs, 1100 benign lesions	ANN	Accuracy: 96.8%^d^; Sensitivity: 38%; Specificity: 99.5%^d^	3

*Abbreviations*, *AK* Actinic keratosis, *ANN* Artificial neural network, *AUROC* Area under receiver operating characteristic, *BCC* Basal cell carcinoma, *CSCC* Cutaneous squamous cell carcinoma, *k-NN* k-nearest neighbors, *MM* Malignant melanoma, *NMSC* Non-melanoma skin cancer, *MSVM* Multiclass support vector machine, *SK* Seborrheic keratosis

^a^Quality rating modified from Simel and Rennie [12]

^b^Competitive set included both benign and malignant lesions

^c^Study tested multiple classifiers; only the classifier that achieved the highest performance has been reported

^d^Figures are derived from confusion matrix values and represent pooled BCC and CSCC classification in studies that tested both

^e^Total test set was derived from three different datasets (“Asan,” “Hallym,” “Edinburgh”), one of which was chronologically assorted and partitioned such that the oldest 90% of images were used for training and the newest 10% for testing [23]. However, number of lesions was provided only for the unpartitioned Asan dataset. Thus, we have estimated the total number of test lesions as 10% of the individual lesion classes in the unpartitioned Asan dataset plus all lesions in the Hallym and Edinburgh datasets

^f^Figure represents approximation from histogram

^g^Training set represents figures provided in a previous study by the experimenters [58]. The classifier has not been retrained [20]

[Table 2. Overview of literature search].

### Skin lesion databases

Twenty exclusively on NMSC, whereas the other 17 studies also included classification of melanoma [14–30]. The size of NMSC test sets ranged from as few as ten lesions [22] to as many as 710 [23]. All studies acquired their images either directly from clinics or from publicly available datasets >composed of clinically-obtained and annotated images, with the exception of nine studies that used images of unverifiable origin from online repositories [14, 19, 21, 22, 24–26, 31, 32]. Among the studies using clinically-obtained image sets, all NMSC images represented biopsy-proven lesions, and seven studies also used exclusively biopsy-proven benign lesions for their competitive sets [15–17, 23, 28, 30, 33].

Eight studies used test sets comprising all lesions examined in a clinic or set of clinics during a specific time frame for which there was any suspicion of malignancy, thus constituting a consecutive sample [15, 17, 30, 34–38]. Two studies, while they did use a set of clinically-obtained images spanning a broad variety of benign lesions suggestive of a consecutive sample, did not explicitly report that this set represented all lesions of suspected malignancy seen in those clinics [39, 40]. The rest of the studies used sets of NMSC lesions and benign mimics chosen by the experimenters. Among the studies using non-consecutive sets, three used actinic keratoses (AKs) as their benign mimic [19, 24, 25], two used seborrheic keratoses (SKs) [16, 41], one used nevi [22], four used SKs and nevi [20, 28, 33, 42], three used AKs, SKs, and nevi [14, 31, 43], one used AKs, SKs, nevi, lentigines, and poromas [18], two used AKs, SKs, nevi, dermatofibromas, and vascular lesions [27, 29], one used AKs, SKs, nevi, dermatofibromas, lentigines, warts, and vascular lesions [23], two used SKs, nevi, and psoriasis [44, 45], one used SKs, nevi, psoriasis, eczema, and seborrheic dermatitis [46], one used SKs, bullae, and shingles [21], one used eczema and impetigo [26], one used hypersensitivity vasculitis and discoid lupus erythematosus [32], and six used benign lesions of unspecified type [47–52]. The three studies that qualified for review by using only AKs as their benign class did so incidentally, as they had designed their experiments as tests of classification between malignancies, in which they counted this pre-cancerous lesion as a true cancer alongside melanoma, CSCC, and BCC [19, 24, 25].

### Methods of feature extraction

Fourteen studies used classifiers trained to detect known dermoscopic features of NMSC lesions. Features of interest included telangiectasia and other vascular features [34, 36, 39, 40, 48, 50–52], semitranslucency [35, 38–40], pink blush [38, 40], ulceration [40, 49], blue-grey ovoids [40, 47], dirt trails [37, 40], scale and scale-crust [41], purple blotches [40], and pale areas [40]. Four studies also incorporated classification based on anatomic location of the lesion and patient profile characteristics including age and gender [28, 39, 40, 51]. Three of these studies also included lesion size [39, 40, 51], two included patients’ geographic location [39, 40], one included ethnicity [40], and one included whether the patient had noted a change in the lesion and whether the patient was concerned about the lesion [39]. The 25 studies that did not use specific features instead utilized global analysis to extract color and texture features of the entire lesion area [14–33, 42–46].

Five studies featured fully automated analysis of raw pixel data with no preprocessing, which they accomplished by using a model that was pre-trained with over 1 million images [16, 18, 23, 44, 45]. Eleven studies involved some amount of preprocessing but achieved automated segmentation of lesion and feature borders [20–22, 27, 29, 30, 37, 39, 42, 49, 51]. Eight studies did not provide sufficient information regarding preprocessing methods to assess the degree of automation [14, 19, 25, 26, 28, 31, 32, 40]. The remaining 15 studies relied on manually outlined lesion or feature borders.

### Methods of classification

The most common ML method was the artificial neural network (ANN), being used in several variations by 20 studies [14, 16, 18, 23, 25, 27–29, 32–37, 40, 44–46, 49, 51]. Variants of the decision tree, namely the decision forest classifier and random forest classifier, were used by four studies [17, 48, 50, 52]. Logistic regression, a linear classification model that may be considered among the simplest forms of ML, was used by four studies [38, 39, 41, 47]. Seven studies used multiclass support vector machines [15, 19, 24, 26, 31, 42]. One study used a k-nearest neighbors algorithm [43], one used a hybrid model of MSVM and k-NN classifiers [21], one used a unique model of layered linear classifiers [20], and one used a hybrid model of linear and quadratic classifiers [30].

Thirty-five of the studies used separate image sets for training and testing or employed cross-validation. The remaining four studies had overlap between training and test sets and thus cannot be considered blinded or independent experiments [41, 47, 48, 50]. Four studies, while they did employ cross-validation, used only images of BCCs that contained specific features of interest that their classifiers were designed to detect, rendering the experiments non-independent [34, 36, 37, 49]. These findings are reflected in the quality ratings shown in Table 2 and the QUADAS-2 summary in Table 3.

**Table 3:** QUADAS-2 summary

	Risk of Bias				Applicability Concerns		
	Patient Selection	Index Test	Reference Standard	Flow and Timing	Patient Selection	Index Test	Reference Standard
Abbas, 2016 [14]	High	Low	High	Low	Low	High	High
Ballerini, 2012 [43]	High	Low	Low	Low	Low	Low	Low
Chang, 2013 [15]	Low	Low	Low	Low	Low	Low	Low
Cheng, 2011 [34]	Low	Low	Low	Low	High	High	Low
Cheng, 2012 [36]	Low	Low	Low	Low	High	High	Low
Cheng, 2013 [37]	Low	Low	Low	Low	High	High	Low
Cheng, 2013 [40]	Unclear	Low	Low	Low	Low	Low	Low
Choudhury, 2015 [24]	High	Low	High	Low	High	High	High
Chuang, 2011 [33]	High	Low	Low	Low	Low	Low	Low
Dorj, 2018 [25]	High	Low	High	Low	Low	High	High
Esteva, 2017 [16]	High	Low	Low	Low	Low	Low	Low
Ferris, 2015 [17]	Low	Low	Low	Low	Low	High	Low
Fujisawa, 2018 [18]	High	Low	Low	Low	Low	High	Low
Guvenc, 2013 [47]	High	High	Low	Low	High	High	Low
Han, 2018 [23]	High	Low	Low	Low	Low	High	Low
Immagulate, 2015 [31]	High	Low	High	Low	Low	High	High
Kefel, 2012 [49]	High	Low	Low	Low	High	High	Low
Kefel, 2016 [38]	Low	Low	Low	Low	Low	Low	Low
Kharazmi, 2011 [48]	High	High	Low	Low	High	High	Low
Kharazmi, 2016 [50]	High	High	Low	Low	High	High	Low
Kharazmi, 2017 [51]	High	Low	Low	Low	Low	Low	Low
Kharazmi, 2018 [52]	High	Low	Low	Low	Low	Low	Low
Lee, 2018 [29]	High	Low	Low	Low	Low	High	Low
Maurya, 2014 [19]	High	Low	High	Low	High	High	High
Mishra, 2017 [39]	Unclear	Low	Low	Low	Low	Low	Low
Møllersen, 2015 [30]	Low	Low	Low	Low	Low	Low	Low
Shakya, 2012 [41]	High	High	Low	Low	High	High	Low
Shimizu, 2014 [20]	High	Low	Low	Low	Low	High	Low
Shoieb, 2016 [26]	High	Low	High	Low	Low	High	High
Stoecker, 2009 [35]	Low	Low	Low	Low	Low	High	Low
Sumithra, 2015 [21]	High	Low	High	Low	High	High	High
Upadhyay, 2018 [27]	High	Low	Low	Low	Low	Low	Low
Wahab, 2003 [32]	High	Low	High	Low	Low	High	High
Wahba, 2017 [22]	High	Low	High	Low	Low	High	High
Wahba, 2018 [42]	High	Low	Low	Low	Low	Low	Low
Yap, 2018 [28]	High	Low	Low	Low	Low	High	Low
Zhang, 2017 [44]	High	Low	Low	Low	Low	Low	Low
Zhang, 2018 [45]	High	Low	Low	Low	Low	Low	Low
Zhou, 2017 [46]	High	Low	Low	Low	Low	Low	Low

*Abbreviation*, *QUADAS-2* The Quality Assessment of Diagnostic Accuracy Studies [13]

### Diagnostic accuracy

All included studies reported at least one NMSC-specific classifier performance metric or provided results in the form of a graph or confusion matrix from which this information could be determined. The most commonly used metric of classifier performance was area under the receiver operating characteristic (AUROC) curve, which was reported by 17 studies [14, 16, 23, 34–38, 40–42, 47–52]. AUROC values ranged from 0.832 [52] to 1 [41], with both extremes reported in studies of BCC-specific classification.

Eleven studies reported accuracy [19, 22, 24–28, 31, 33, 46, 47], four studies provided a confusion matrix from which accuracy could be calculated [43–46], and one study provided a graph from which accuracy could be estimated but did not provide an exact numerical fig. [39]. Sensitivity was reported or derivable from a confusion matrix in 22 studies [14, 15, 17, 18, 20, 22, 23, 25–30, 32, 33, 42–46, 51, 52], and 15 of these studies also provided specificity data [14, 22, 23, 25, 26, 28, 30, 33, 42–46, 51, 52]. The highest accuracy, specificity, and sensitivity were reported by two BCC studies that achieved 100% for each of these metrics [22, 42]. Notably, one of these studies used a test set of only ten images, the smallest of all the included studies [22]. Another study achieved 100% sensitivity in combined BCC and CSCC classification, however the corresponding specificity of this test was 12%, the lowest of all the included studies [30]. The lowest accuracy and sensitivity were 72% [39] and 38% [46], respectively, both of which represented BCC-specific classification. Moreover, one study reported only F-measure, which is a metric that reflects both precision and recall, with a value of 0.581 for CSCC-specific classification [21].

Six studies tested the performance of their classifier against dermatologists [15–18, 23, 46]. Two of these studies were not focused primarily on NMSC classification and thus did not include NMSC lesions in the test [17, 46]. Esteva et al. conducted two experiments comparing the performance of an ANN classifier against a group of 21 board-certified dermatologists using sets of 135 biopsy-proven lesion images (65 NMSC, 70 SK). In the first experiment, the dermatologists were asked whether or not they would biopsy the lesion and, in the second experiment, if they thought the lesion was benign or malignant. The results of these trials were presented as receiver operating characteristic (ROC) curves, and in both cases, the performance of the majority of the dermatologists fell beneath the classifier ROC curve, indicating that the classifier had outperformed them on average [16]. Similarly, Han et al. tested an ANN classifier against 16 board-certified dermatologists using a set of 460 benign and malignant lesions, including 25 BCCs and 25 CSCCs. The dermatologists were asked to select the correct diagnosis for each lesion from 12 possible choices. Results were presented as disease-specific ROC curves, which again showed lower average performance by the dermatologists compared to the ANN classifier for both BCC and CSCC classification [23]. Chang et al. compared a MSVM classifier against the performance of 25 board-certified dermatologists using a set of 769 benign and malignant lesions, which included 110 BCCs and 20 CSCCs. Dermatologists were able to correctly classify 88.2% of BCCs and 85% of CSCCs as malignant, compared to the MSVM classifier, which correctly classified 90% of BCCs and 80% of CSCCs [15]. Lastly, Fujisawa et al. compared the performance of an ANN classifier against 13 board-certified dermatologists by having each dermatologist classify a different set of 140 images randomly selected from a set of 1142 lesion images spanning 14 diagnoses, including 249 BCCs and 189 CSCCs. The ANN classifier was able to correctly classify 80.3% of the BCCs and 82.5% of the CSCCs, whereas the dermatologists correctly classified 64.8% of BCCs and 59.5% of CSCCs [18]. Of note, none of these studies included *P*-values for these comparisons, so it is uncertain if the reported differences in performance relative to dermatologists were statistically significant.

## Discussion

The use of AI is rapidly expanding in dermatology and society in general. As the world population and average human life span continue to rise, access to rapid skin cancer screening is becoming increasingly important., Digital image-based ML models present an intuitive and promising means of extending the reach of dermatologists to meet this growing need. Though computerized analysis of skin lesions has been an active area of research for nearly 30 years, NMSC has been relatively understudied. Indeed, perhaps the most striking finding of this review is the relative paucity of NMSC-specific image analysis research. In contrast, a systematic review of automated melanoma detection that was conducted 10 years ago and restricted to only dermoscopic literature identified 30 studies [8], nearly the same number as in the present review.

Most of the studies in this review structured their experiments as computational proofs of concept and thus did not adequately account for criteria that are traditionally used to evaluate diagnostic studies. In particular, 29 studies did not use a consecutive sample [14, 16, 18–29, 31–33, 41–52], four studies were not independent [34, 36, 37, 49], four studies were neither blinded nor independent [41, 47, 48, 50], and nine studies used reference standards of uncertain validity [14, 19, 21, 22, 24–26, 31, 32]. Only seven studies used exclusively biopsy-proven lesions as their benign mimics, which can only be accomplished by strictly including lesions that are clinically suspicious for malignancy [15–17, 23, 28, 30, 33]. While this methodology can be seen as contributing to a higher level of evidence, its absence does not harm validity in studies using lesions that are benign only by clinical examination, given that biopsy of such lesions in the absence of symptoms is not only inconsistent with standards of care but also unethical. Three studies included only AKs as a benign mimic [19, 24, 25]. While this lesion is technically benign, it is considered a precursor of CSCC [53]. This same limitation affected several studies reporting NMSC-specific performance metrics in experiments that included melanoma in the test set [14, 18–21, 23–26, 28, 29, 35]. In this case, there is no clinical value in counting a melanoma that has been classified as non-NMSC as a “true negative” unless it is concurrently classified as a malignancy. In contrast, two studies, while they did include melanoma in their test sets, designed their classifiers for binary classification of lesions as benign or malignant rather than multiclass classification, thus avoiding this issue [15, 27]. Another two studies presented multiclass classifiers that were able to achieve 95% or greater sensitivity for both melanoma and NMSC detection [30, 42].

Another important limitation in most of the reviewed studies was the need for extensive preprocessing of images. Lesion border detection was a particularly challenging preprocessing step, which most of the studies performed by hand. Only five studies presented a fully automated model, bypassing the need for any hand-crafted features by using a classifier that was pre-trained with an image set that was orders of magnitude larger than that of the other studies [16, 18, 23, 44, 45]. This sort of data-driven solution is perhaps the most promising approach to the challenge of variable clinical presentations and imaging conditions.

A few studies were robustly designed as blinded, independent experiments using consecutive samples and thus attained the highest level of evidence possible for studies of their size [15, 17, 30, 35, 38, 40]. However, one of these studies was primarily focused on melanoma detection and used only 14 NMSC lesions in its test set, reducing its applicability [17]. Most studies reported accuracy metrics greater than 90%, which is higher than the average clinical accuracy of dermatologists published in large, multicenter studies [54, 55]. The most compelling evidence to this end was provided by the four studies in this review that directly tested NMSC classifiers against groups of dermatologists, demonstrating higher accuracy by the ML models [15, 16, 18, 23].

The major limitation of all the studies in this review is that they were conducted entirely in silico. Until a NMSC detection system is designed with complete human-computer interface and tested in actual clinical trials as has been done for melanoma in the case of MelaFind [56, 57], the applicability of these classifiers remains theoretical. Moreover, only one of the included studies reported the run times of its model, which were 0.58 s and 3.02 s for BCC and CSCC classification, respectively [27]. While these values are favorable, the computational times of other classifiers cannot necessarily be assumed to be comparable. Given that it is not uncommon for a patient to present to a dermatologist with multiple NMSC lesions and benign mimics in a single visit, it is critical for computer-aided diagnostic systems to perform not only accurately but also rapidly.

## Conclusion

The overall quality of evidence for the diagnostic accuracy of digital image-based ML classification of NMSC is moderate. While nearly all included studies reported high diagnostic performance, the majority had considerable methodological limitations. Common issues included the use of non-consecutive samples, overlapping training and test sets, and non-biopsy proven reference standards. Nevertheless, several studies did provide high level evidence for ML classifiers capable of accurately discriminating NMSC from benign lesions in silico. Further research is needed to test the viability of these models in a clinical setting.

## Additional file

Additional file 1: PRISMA checklist. (DOC 66 kb)

## Abbreviations

AI: Artificial intelligence
AK: Actinic keratosis
ANN: Artificial neural network
AUROC: Area under receiver operating characteristic
BCC: Basal cell carcinoma
CSCC: Cutaneous squamous cell carcinoma
k-NN: K-nearest neighbors
ML: Machine learning
MM: Malignant melanoma
MSVM: Multiclass support vector machine
NMSC: Nonmelanoma skin cancer
PRISMA: Preferred Reporting Items for Systematic Reviews and Meta-Analyses
QUADAS-2: Quality Assessment of Diagnostic Accuracy Studies, 2nd edition
ROC: Receiver operating characteristic
SK: Seborrheic keratosis
